# Tick-borne encephalitis virus variants drive distinct TCR repertoire alterations

**DOI:** 10.3389/fimmu.2025.1663781

**Published:** 2025-12-18

**Authors:** Maria A. Salnikova, Ksenia K. Tuchynskaya, Anastasia A. Minervina, Mikhail V. Pogorelyy, Egor V. Okhezin, Galina G. Karganova, Ilgar Z. Mamedov, Yuri B. Lebedev

**Affiliations:** 1 Department of Genomics of Adaptive Immunity, Shemyakin-Ovchinnikov Institute of Bioorganic Chemistry Russian Academy of Sciences (RAS), Moscow, Russia; 2 Department of Molecular Technologies, Institute of Translational Medicine, Pirogov Russian National Research Medical University, Moscow, Russia; 3 Department of Immunology, Faculty of Biology, Lomonosov Moscow State University, Moscow, Russia; 4 Federal State Budget Scientific Institution (FSBSI) “Chumakov Federal Scientific Center for Research and Development of Immune-and-Biological Products of Russian Academy of Sciences (FSC R&D IBP RAS)“, Institute of Poliomyelitis, Moscow, Russia; 5 Institute of Translational Medicine and Biotechnology, Sechenov First Moscow State Medical University, Moscow, Russia

**Keywords:** T cell, T cell receptor, TCR sequencing, TCR clusters, tick-borne encephalitis (TBE), TBEV infection, *Orthoflavivirus*

## Abstract

**Background:**

T cells play a crucial role in the adaptive immune response against acute virus infections. The extensive diversity of T cell receptors (TCRs) presents a complex challenge for understanding its implications in immune responses. Investigating the dynamics of the immune response to acute virus infection is inherently more complex compared to studying vaccine responses, but it offers a more comprehensive view on the subject matter.

**Methods:**

Therefore, we used an immunosequencing approach to investigate acute viral infections in a murine model system. Specifically, we analyzed the TCRβ repertoire to identify dissimilarities in the immune response of BALB/c mice against different variants of tick-borne encephalitis virus (TBEV), which differ by a few amino acid substitutions and are derived from the same parental strain.

**Results:**

We identified numerous TCRβ clonotypes that responded to the infection. Furthermore, we observed differences in the magnitude of the T cell response depending on the virulence of either the TBEV variant or the immature TBEV particles. Interestingly, regardless of the viral variant, we observed a shift towards CD8+ T cells among TBEV-associated T cells. Additionally, our findings revealed that TBEV induced massive alterations in through the most represented T cell clones, leading to TCRβ repertoire rearrangement.

**Conclusion:**

We were able to identify sequence similarities among TBEV responding clones in mice infected with different virus variants. These findings provide valuable insights into the dynamics of T cell responses during acute viral infections and highlight the importance of studying TCR diversity for an in-depth understanding of the immune response.

## 1 Introduction

Immunosequencing reveals huge amounts of information about specific immunity and hidden biological mechanisms (1–4). Numerous studies have been conducted on the topic of human immunity, for instance dynamics of the TCR repertoire during lifespan (5–7), the identification of T cell clones linked to autoimmune disorders, such as ankylosing spondylitis (8), the monitoring of TCR repertoire during persistent infections (9), the investigation of vaccine impacts (10–13), all contribute to a more comprehensive understanding of the principles governing antigen recognition and the potential clinical applications (14–16).

TCR sequencing has become a powerful approach for investigating adaptive immunity, offering unique insights, particularly in the context of viral disease research. This approach enables detailed characterization of how TCR repertoire architecture changes during antiviral responses, including the identification of clonal expansions at the level of individual T cell clones (9). Moreover, TCR sequencing facilitates comparative analyses across individuals and/or populations, allowing to distinguish shared (“public”) from unique (“private”) clones (17, 18) Finally, by clustering TCR sequences, this approach can reveal conserved motifs that point to previously unknown viral epitopes, thereby advancing our understanding of virus-specific T cell recognition (19, 20).

Rapid drug and vaccine industry development requires up-to-date murine models, because mice still remain a basic issue for pre-clinical trials (21). In the field of immunology, murine models of acute viral infections are a useful tool for investigating the TCR repertoire. These models provide controlled experimental conditions to track alterations in the immune system during viral diseases. TCR sequencing in murine models enables to profile immune cells infiltrating affected organs or secondary lymphoid organs (22). By determining murine smallpox vaccine-associated T cells, it becomes possible to create a diagnostic classifier of the TCR repertoires (23). Analysis of murine TCR repertoire data plays a crucial role in distinguishing between chronic and acute infections (24).

Tick-borne encephalitis (TBE) is one of the most important viral zoonosis diseases in Europe and Russia (25). The causative agent of TBE is *Orthoflavivirus* encephalitis (26). The Orthoflavivirus genome consists of a single positive-sense RNA molecule that encodes 3 structural [capsid protein (C), pre-membrane protein (prM), and envelope protein (E)] and 7 non-structural proteins (NS1, NS2A, NS2B, NS3, NS4A, NS4B, NS5). The E protein forms the outer surface of the virion and is involved in such important processes as attachment and penetration into the host cell, also it is the main target for antiviral neutralizing antibodies (27). Certain point mutations in the sequence of the E protein affect neuroinvasiveness and the virus’s ability to spread among host cells (28), influencing the adsorption of virions to glycosaminoglycans, which serve as low-affinity receptors for Orthoflaviviruses. During reproduction, Orthoflavivirus virions first assemble into immature, non-infectious particles on the endoplasmic reticulum membrane. These particles must undergo cleavage of the N-terminal ‘pr’ peptide on the M protein by the cellular furin protease to become infectious. As a result, peptide antigens of immature prM can be lost. However, this maturation process is often inefficient, resulting in the release of a mixture of mature virions, immature particles, and partially-mature particles. The delayed disease and mortality might be mediated by residual mature virions. This heterogeneity can influence the immune response during infection and further mortality (29).

Transmission of tick-borne encephalitis virus (TBEV) occurs through tick bites or consumption of unpasteurized milk from infected animals. The incidence of TBE cases in Russia and Europe has increased significantly in recent years (30–32). Multiple factors can influence the severity of the disease, such as the specific virus variant and virus dose (33, 34), duration and site of the tick bite, host genetic traits, and the immune response to the infectious agent (35). Within this expansive region, TBEV manifests in various subtypes, including European, Siberian, Far Eastern, Baikalian, Himalayan subtypes, and others (36). These subtypes differ in terms of severity and clinical characteristics (25).

In this study, we used an immunosequencing approach to analyze the immune response in mice infected with different variants with point mutations of tick-borne encephalitis virus (TBEV) strain EK-328 Siberian subtype as well as immature virus (37, 38). The selection of TBEV variants was based on an analysis of their functional traits and structural characteristics, specifically focusing on the envelope (E) as well as non-structural proteins.

Overall, we detected 820 TBEV-associated T cell clones. Amino acid sequences of TBEV-associated CDR3 TCRβ are clustered across mice and variants. Our study provides valuable knowledge about T cell immune response during the infection with different TBEV variants.

## 2 Materials and methods

### 2.1 Cells and viruses

The pig embryo kidney (PEK) cell line was maintained at 37 °C in Medium 199 (Chumakov FSC R&D IBP RAS, Russia), supplemented with 5% fetal bovine serum (FBS, Gibco) (37) line was used for the virus titration and neutralization assay. Three variants from TBEV strain EK-328 were used in this study. The original TBEV strain EK-328 isolated from ticks *Ixodes persulcatus* (GenBank ID DQ486861.1) (37), which contains additional several synonymous substitutions (39), was adapted to the mouse brain through a maximum of 10 passages and two passages in the PEK cell line (**Supplementary Figure 1**). Variant M (original name clone 718/574) (37) with two additional synonymous mutations (39) was obtained from parental strain EK-328 after 17 passages in the *H. marginatum marginatum* ticks, 5 additional passages through the mouse brain and one passage in the PEK. Clone 991/58 was derived from variant M by plaque purification after the passage in the PEK cell culture detailed in previous work (37, 40) and subsequently underwent additional cloning (**Supplementary Figure 1**). All nucleotide substitutions in the genome of the M variant and clone 991/58 compared to the parental strain EK-328 are listed in **Supplementary Table 1**.

In addition to TBEV virus variants, the study also investigated an immature virus. It was obtained by the addition of 20 mM of NH4Cl at one and five hours of EK-328 infection in the PEK cell line (38).

### 2.2 Virological characteristics of the studied virus

#### 2.2.1 Quantification of genome containing particles by qRT-PCR

Reverse transcription was performed with M-MLV reverse transcriptase (Promega, USA) according to the manufacturer’s protocol. Real-time PCR was carried out on DNA Engine analyzer (BioRad) using RT-PCR kit (Syntol, Russia). As internal control for efficiency of RNA reverse transcription of Poliovirus strain Sabin I was added in the same concentration to each sample before extraction. Reverse transcription was performed with primers Pow-TBE-3’: 5’- AGCGGGTGTTTTTCCGAGTC-3’ for TBEV and PVR1: 5’-CGAACGTGATCCTGAGTGTT-3’ for Poliovirus. Polymerase chain reaction was performed with primer for TBEV R-TBE: 5’-ACACATCACCTCCTTGTCAGACT -3’, F-TBE: 5’-GGGCGGTTCTTGTTCTCC-3’ and probe TBE: 5’-(FAM*)-TGAGCCACCATCACCCAGACACA-(BHQ1*)-3’ for 3’-end of TBEV genome and by using standards with a known concentration of TBEV RNA described earlier (Gmyl et al., 2003). For internal control PVR1, PVL1: 5’-GGCAGACGAGAAATACCCAT -3’ and probe PVP: 5’- (R6G*)-TTGATTCATGAATTTCCTTCATTGGCA-(BHQ1)-3’were used for qRT-PCR (32). The concentration was expressed as the logarithm of the viral genome copy number (GCN) per ml (log10GCNmL). Experiments were repeated three times for each sample to calculate an average value.

#### 2.2.2 Quantification of infectious virus

Virus was quantified using a plaque assay conducted in 24-well plates with PEK cells under a 1.26% methylcellulose (Sigma, USA) overlay as described earlier (33). Plaques were visualized by staining with 0.4% gentian violet dye (Sigma, USA) and subsequently counted. The number of plaques was expressed as log_10_PFU per mL. Two parallel replicates were conducted for each sample.

#### 2.2.3 Viral infection characteristics

The neuroinvasiveness index for female BALB/c mice was estimated as the ratio between the number of infectious viral particles determined by plaque assay in PEK cells (PFU) and the 50% lethal dose (LD_50_) of the virus after subcutaneous (s/c) inoculation, calculated according to the Kerber method (41) **Table 1**. Virulence is presented as a ratio between the genome copy number (GCN) and the 50% lethal dose (LD_50_) of the virus after subcutaneous (s/c) inoculation as described previously (42) **Table 1**.

**Table 1:** Virological characteristics of the studied virus variants.

Virus variant	Plaque size, mm^A^	Amino acid substitutions (compared with the parental strain EK-328)^B^	Virulence index	Neuroinvasiveness index	GCN to infectious titer ratio
			log_10_(GCN/LD_50_)	log_10_(PFU/LD_50_)	log_10_(GCN/PFU)
EK	6-8	parental variant	6.5 ± 0.6	2.9 ± 0.5	3.6 ± 0.4
991/58	6-8	(NS2a) Lys52 →Asn (NS4a) Ser22 → Gly (NS4a) Arg41 → Lys	6.5 ± 0.5	3.1 ± 0.5	3.4 ± 0.1
M	pp^C^D: 5-8 = 1: 100^D^	(E) Glu122→ Gly (E) Thr426→ Ile/ Thr (NS2a) Lys52 →Asn (NS4a) Ser22 → Gly (NS4a) Arg41 → Lys	10.4 ± 0.6	6.5 ± 0.5	3.9 ± 0.4
IM	6-8	no substitutions compared to EK	8.3 ± 0.6	2.1 ± 0.6	6.2 ± 0.4

A. Size of plaques in PEK cells at 7 d.p.i. The percentage of each plaque size is shown in brackets.

B. Virus protein with a mutation is shown in parenthesis.

C. Pinpoint plaque.

D. The ratio between small and large plaques due to the genetic heterogeneity of M variant.

### 2.3 Experiments in mice

#### 2.3.1 Animals and viral infection

As a murine model, we have selected the BALB/c strain of mice due to its increased susceptibility to TBEV (38, 43). In experiments, female BALB/c mice (8 weeks old) (FSBI “Scientific Center of Biomedical Technologies” of Russian Academy of Sciences, “Stolbovaya” branch) were divided into groups of 10 and injected subcutaneously with 100 μl of virus suspended in Earle’s balanced salt solution (199 medium) (Chumakov FSC R&D IBP RAS, Russia) at an infectious dose of approximately 6 log^10^PFU per mouse. The dose of the immature virus sample was equalized by the number of GCN relative to mature EK-328.

This study was carried out according to the international guidelines for the handling of laboratory animals (CIOMS Recommendations, 1985, Directive 2010/63/EC and Annex A of the European Convention ETS No. 123). The study protocol was approved by the Ethics Committee of the FSBSI “Chumakov FSC R&D IBP RAS (protocol #29012018 from 29.01.2018).

#### 2.3.2 Blood sample processing

The blood from the mice was collected from the submandibular vein into Eppendorf tubes containing 10% (v/v) 0.5 M EDTA (Sigma, USA) at -7 days and 7 days post-virus inoculation. Each blood sample was divided into two aliquots of 100–125 μl. Subsequently, 900 μl of TRIzol (Invitrogen, USA) was added to each aliquot using active pipetting. Subsequently, the samples were frozen at -70°C until further analysis.

#### 2.3.3 Whole body mice perfusion

Each mouse was anesthetized using 100 μl of Zoletil 100 (Verbac S.A., France) diluted to a 1:10 ratio. After anesthesia, the mouse was secured in a supine position. Perfusion was performed with 5 mL of 0.9% NaCl solution by introducing the solution into the posterior aspect of the left ventricle. After puncturing the left ventricle with a needle, a small incision was made in the right atrium to facilitate perfusion (44). The quality of perfusion was assessed by observing the lightening of the color of the brain and liver.

#### 2.3.4 Spleen sample processing

Spleen was collected at 7 days after virus inoculation to the Eppendorf with RPMI 1640 solution (Chumakov FSC R&D IBP RAS, Russia) and 2% fetal bovine serum (FBS) (Gibco, Invitrogen, USA), 2mM L-Gln (Chumakov FSC R&D IBP RAS, Russia), and 0,1% gentamicin (Complete medium). Each spleen was washed several times with a complete medium, eliminated fat and gently homogenized without loss of splenocyte functionality. Splenocytes were precipitated by centrifugation 10’ at 400g on 4 °C in Eppendorf 5804R. Erythrocytes removal was carried out using ACK Lysing buffer (Gibco, Invitrogen, USA) according to manufacturer’s instructions. The cells were counted in a hemocytometer and diluted to 10^6^/mL. CD4^+^ and CD4^-^ populations were isolated from splenocytes using magnetic beads (Mouse Dynabeads CD4^+^, Invitrogen, USA) according to the manufacturer’s protocol. 500 ml of Trizol was added directly onto CD4^+^ and CD4^-^ cells populations(Invitrogen, USA). The samples were frozen at -70°C until further investigation.

#### 2.3.5 Infectious virus detection in the brains of infected mice

To detect the virus in the brain tissue of 8-week BALB/c mice 7 days after infection 10% brain suspension underwent a single passage in PEK cell culture to amplify the virus. The presence of virus in the culture fluid was subsequently determined using (ELISA) commercially available kits (Vector-Best, Novosibirsk, Russia).

#### 2.3.6 50% plaque reduction neutralization test

Blood samples were collected from mice by decapitation on day seven after infection with the viruses under study. The blood was left at +4 °C for 1 hour, after that it was centrifuged at 300 g for 10 minutes. The serum was aliquoted and stored at -20 °C until further analysis. The plaque reduction neutralization test (PRNT50) was performed on PEK cell monolayers in 24-well following the protocol described previously (38). The proceeding steps were identical to those used for the plaque assay. Each experiment included negative and positive murine sera controls with established antibody titers. Neutralizing antibody (NAb) titers were calculated according to the modified Reed and Muench method (45).

#### 2.3.7 TCRβ cDNA library preparation and sequencing

Total RNA was isolated from all samples using TRIzol reagent. Complementary DNA (cDNA) synthesis was performed utilizing 5’-RACE template switch technology, which included double-barcoding. Each cDNA molecule was labeled with a sample barcode and Unique Molecular Identifiers (UMIs). This approach helped not only to introduce universal primer binding sites, but to avoid contamination among samples and to count the amount of start mRNA molecules (46). Mouse TCR profiling kit (MiLaboratory LLC) was used for cDNA TCRβ libraries preparation. Bulk TCRβ sequencing was performed on Illumina HiSeq platform with 2X150 bp sequencing length. Detailed information regarding the procedure can be found in SI Appendix.

#### 2.3.8 Raw sequencing data analysis

Raw data was processed during two stages. At the first stage PCR-errors were eliminated and row reads were clustered by UMIs (unique molecular identifiers) using open-source software tool MiGEC (47). At the second stage the MiGEC output were processed by MiXCR (48) to correct PCR and sequencing errors and to extract CDR3 TCRβ clonotypes with marked V, D, and J segments.

#### 2.3.9 Identification of changed clonotypes by edgeR

We used EdgeR package (49) to identify the significantly expanded clonotypes after infection with TBEV as previously described (12). For each timepoint we collected and analyzed two biological replicates of whole blood samples. Following the edgeR manual TMM-normalization and trended dispersion estimates was performed. To identify clones significantly expanded between pairs of timepoints we used an exact test based on the quantile-adjusted conditional maximum likelihood (qCML). We assigned clonotype to the group of significantly expanded, if its log_2_ fold change estimate log_2_FC>7 and its p-value after multiple testing correction was lower than 0.05.

#### 2.3.10 Phenotyping of significantly expanded clonotypes

To assign phenotype to expanded clones we used repertoires from two fractions of splenocytes (CD4^+^ fraction and CD4^-^ fraction) for each mouse individually. The circulating T-cells are either CD4^+^ or CD8^+^, thus here we use CD4^-^ fraction as a proxy for CD8^+^ fraction. For each clone we calculated its concentration in CD4^-^ fraction to concentration in CD4^+^ fraction ratio. We assigned clone a CD8 phenotype if this ratio were more than 3, otherwise the clone was identified as CD4^+^. The expanded clones are abundant in both blood and spleen samples after the TBEV infection, therefore there were no expanded clones with undetermined phenotype. Detailed information regarding the procedure can be found in **Supplementary Figure 3**.

#### 2.3.11 Graph analysis of the expanded clonotypes

The similarity network was processed by the Igraph package (50). To create similarity network TBEV-associated TCRβ CDR3 amino acid sequences from each mouse were combined, additionally V segment and phenotype (CD4^+^/CD8^+^) were added. TCRβ CDR3 amino acid sequences were used as nodes. Edges are aimed to connect nodes with similar CDR3 sequences. An undirected graph object was constructed from a TBEV-associated TCRβ CDR3 amino acid sequences file. The Hamming distance is used to calculate similarities. Edges were created only for TCRβ CDR3 amino acid sequences with Hamming distance 2 or less. Nodes additional information: phenotype (CD4^+^/CD8^+^), TBEV-variant, V-gene were added as attributes. Visualization of the network was conducted using Gephi (51). The ForceAtlas2 algorithm layout was used. Nodes were colored by their TBEV-variants specificity or phenotype, and sized proportionally to their degree centrality.

#### 2.3.12 Additional analysis of the expanded clonotypes

Structural motifs of TCRβ CDR3 amino acid sequences from detected clusters were processed by ggseqlogo R package (52). Each letter is specific to amino acids. The probability for each position was calculated using ggseqlogo R package, conserved amino acids correspond to bigger letters with probability equal to 1 and the variable positions are shown as columns of letters, letter size corresponds to probability.

P_gen_ (probability of generation) is the probability of a certain TCR chain to be generated during V(D)J recombination. P_gen_ integrates the probability of selecting a particular V, D, and J gene, deletions and insertions during V(D)J recombination. The P_gen_ for expanded TCRβ CDR3 amino acid sequences was calculated using OLGA software with murine TCRβ settings (53).

In addition to identified TBEV-associated TCRβ clonotypes we analyzed published data of TCRβ CDR3 amino acid sequences associated with Far Eastern TBEV strain (54). Firstly, the data previously published by Fujii et al. were preprocessed to achieve uniformity in V-segments nomenclature. Afterwards the similarities between this data and TBEV-associated CDR3 amino acid sequences found by us were measured using Hamming distance. As no similarities were found, an additional graph with TCRβ CDR3 amino acid sequences previously published by Fujii et al. were created and combined with existing graph of TBEV-associated clonotypes for improved visualization.

#### 2.3.13 Statistical analysis

Statistical analysis was conducted using the R programming language. The Mann–Whitney U test was employed to compare the quantities of expanded clonotypes. To account for multiple comparisons, resulting p-values were adjusted using the Benjamini–Hochberg (BH) method. The significance of shifts towards CD8^+^ T cells was evaluated using the Wilcoxon Signed-Rank Test.

To identify significant changes in V-segment usage, two-sided t-test p-values were calculated for differences in segment frequencies between TBEV-associated clonotypes and the baseline TCR repertoire at day -7. Given that hypotheses related to specific segments can be considered independent, we applied the Benjamini-Hochberg (BH) procedure with two-sided t-tests to control for multiple testing errors. The false discovery rate during normality testing was maintained at ≤0.05 by setting an upper bound for adjusted p-values at 0.05.

## 3 Results

### 3.1 Characteristics of TBEV infection dynamics in mice

**Figure 1:**
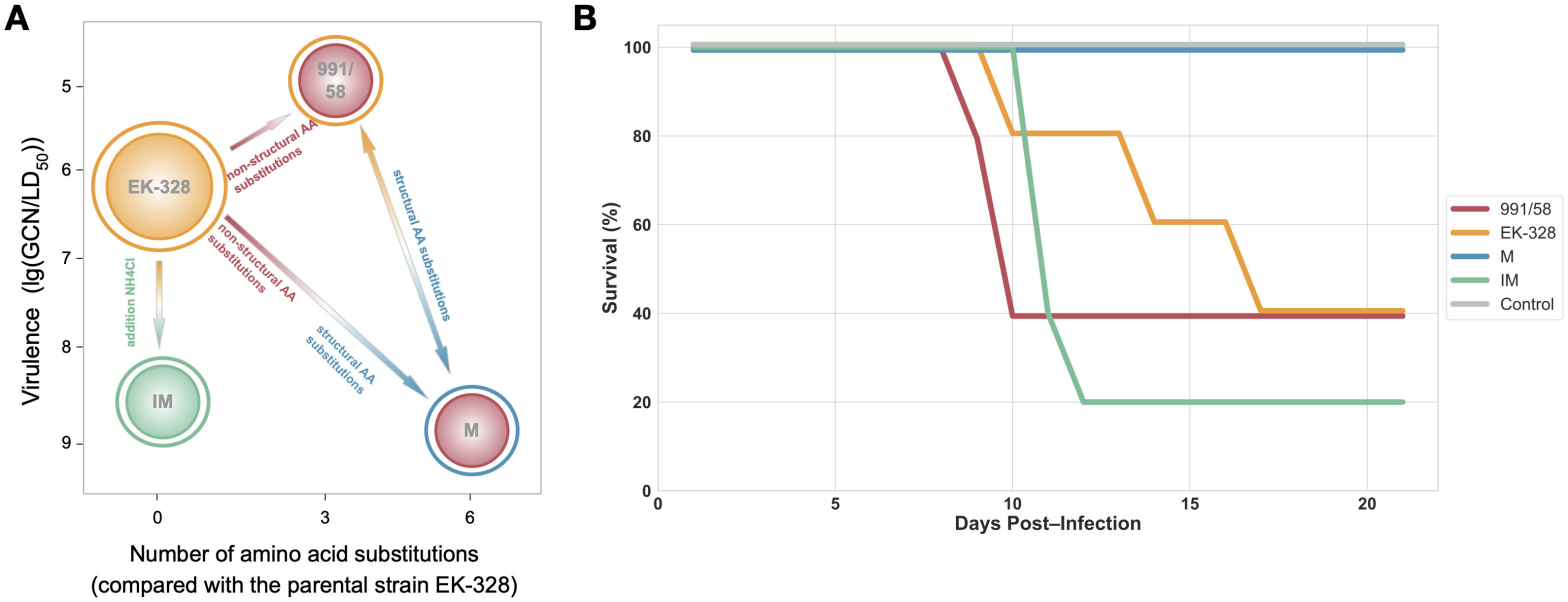
Characteristics of TBEV variants. **(A)** Simplified scheme of TBEV variants relationships. Colors of the outer and inner layer represent structural and non-structural proteins dissimilarity, respectively. IM and EK-328 have no amino acid substitutions, but as EK-328 is a mature variant, it displays cleaved M-protein (yellow), while IM has pr-M (green). EK-328 and 991/58 variants have similar structural proteins sequences (yellow), but differ in sequences of non-structural proteins (red). The M variant has the same sequence of non-structural proteins as 991/58 (red) and is different from all variant substitutions in structural proteins (blue). Y-axis represents virulence of the virus, where 991/58 and EK-328 are the most virulent. This scheme does not represent the passage history **(B)** Kaplan-Meier survival curve for mice infected with different TBEV variants, n=5.

We selected three virus variants derived from the TBEV strain EK-328 (37). These variants included the wild type EK-328 (EK) variant, the M variant, the revertant 991/58 variant. In addition we studied the immature virus form of EK-328 (IM variant). The detailed passage history of these variants is described in the Materials and Methods section. The parental variant EK-328 serves as the precursor to M, which has several amino acid substitutions in protein E as well as additional amino acid substitutions in non-structural proteins (**Figure 1A**). The 991/58 revertant has functional characteristics closer to the parental EK-328 (**Table 1**). The 991/58 variant’s E protein sequence completely coincided with EK-328, but it had similar amino acid sequence mutations in non-structural proteins as the M variant. Immature EK-328 (IM) differs from parental EK-328 only in presence of the uncleaved immature protein prM, distinct surface structure displayed by the majority of viral particles, rendering them non-infectious (**Figure 1A**).

The studied viruses varied in terms of their virulence and neuroinvasiveness. The virulence index shows the ratio of viral genomes identified by PCR to the number of viral particles that cause the death of 50% of animals (i.e. the LD_50_). Therefore, the higher the index, the greater the number of non-infectious viral particles present in the sample. The virulence index for EK-328 and 991/58 variant was lower than for the M variant and the IM virus, indicating the presence of a large number of non-infectious viral particles for these (M and IM) viruses (**Table 1**). The neuroinvasiveness index is the number of infectious virions required to cause 50% mortality in animals administered subcutaneously. Neuroinvasiveness index comparison shows that the M variant has the lowest score, while the residual infectious virus in the IM virus sample has the same neuroinvasiveness index as EK-328 (**Table 1**). The total number of GCN for the studied viruses was approximately the same. GCN to PFU ratio was almost identical for all virus variants except IM virus variant (**Table 1**). Therefore the dose of the immature virus sample was equalized by the number of GCN relative to mature EK-328, other viruses were equalized by the number of log_10_PFU (**Table 2**).

**Table 2:** Characterization of the infection in a given dose of virus.

Virus variant	Infection dose			Mice exhibiting viral presence in brain tissue at 7 days post-infection	Log_10_NAb against test virus in mouse sera at 7 days post-infection, n=3-5	Number of survived animals^C^	Number of animals without disease manifestations^D^
	log_10_PFU_A_	log_10_GCN	LD_50_				
EK	6	9.7	1500	40% (4/10)	2.3 ± 0.5^B^	40% (2/5)	0% (0/5)
991/58	6	9.4	1000	38% (3/8)	2.1 ± 0.3	40% (2/5)	0% (0/5)
M	6	9.5	0,5	0% (0/8)	1.9 ± 0.8	100% (5/5)	100% (5/5)
IM	2.7	8.9	4	0% (0/8)	0	20% (1/5)	0% (0/5)

A. The error in the experimental method was previously determined as 0.5 log_10_PFU (54).

B. Log_10_NAb determined in PRNT50. Mean ± SD.

C. Data by 21 days post infection.

D. Signs of the disease were taken into account with a weight loss of more than 1.5 g, ruffled of mice, intoxication, paresis and paralysis of limbs.

The description of TBEV pathogenesis in terms of temporal progression required independent experimental cohorts. To maintain consistency and comparability among experimental groups, mice were infected with the same virus dose (**Table 2**). By day +7 post-infection, about 40% of the murine subjects inoculated with EK-328 or 991/58 viral variants showed presence of virus particles in the brain tissue (**Table 2**), whereas animals infected with variants M and IM did not exhibit viral presence in the brain on that day. Longitudinal monitoring revealed divergence in outcomes by day 21 post-infection (**Figure 1B**): animals infected with EK-328, 991/58, or IM experienced high mortality, whereas the majority of M-variant-infected mice displayed no clinical manifestations of disease (**Table 2**). It should be noted that for experimental infections with some TBEV strains, such as EK-328, animal mortality is non-linear (55).

To improve the understanding of the adaptive immune response against these TBEV variants a separate experiment was carried out. We infected mice with the same virus dose and measured the quantity of neutralizing antibodies against virus variants (**Table 2**). For EK, 991/58 and M variant, the antibodies level against challenged viruses did not differ, whereas for IM virus the antibody response against EK-328 was significantly reduced. This can indirectly specify the role of TBEV structural proteins and excessive amounts of immature non-infectious viral particles in anti-virus adaptive immune response.

Based on our observations, we distinguished the group of comparison: the highly virulent and neuroinvasive variants (991/58 and EK), and the low-virulent, low-neuroinvasive M variant. We also consider the mature (EK) and immature (IM) form of the same virus separately.

### 3.2 TCR repertoire during TBEV infection

#### 3.2.1 Study design and identification of virus responding clonotypes

**Figure 2:**
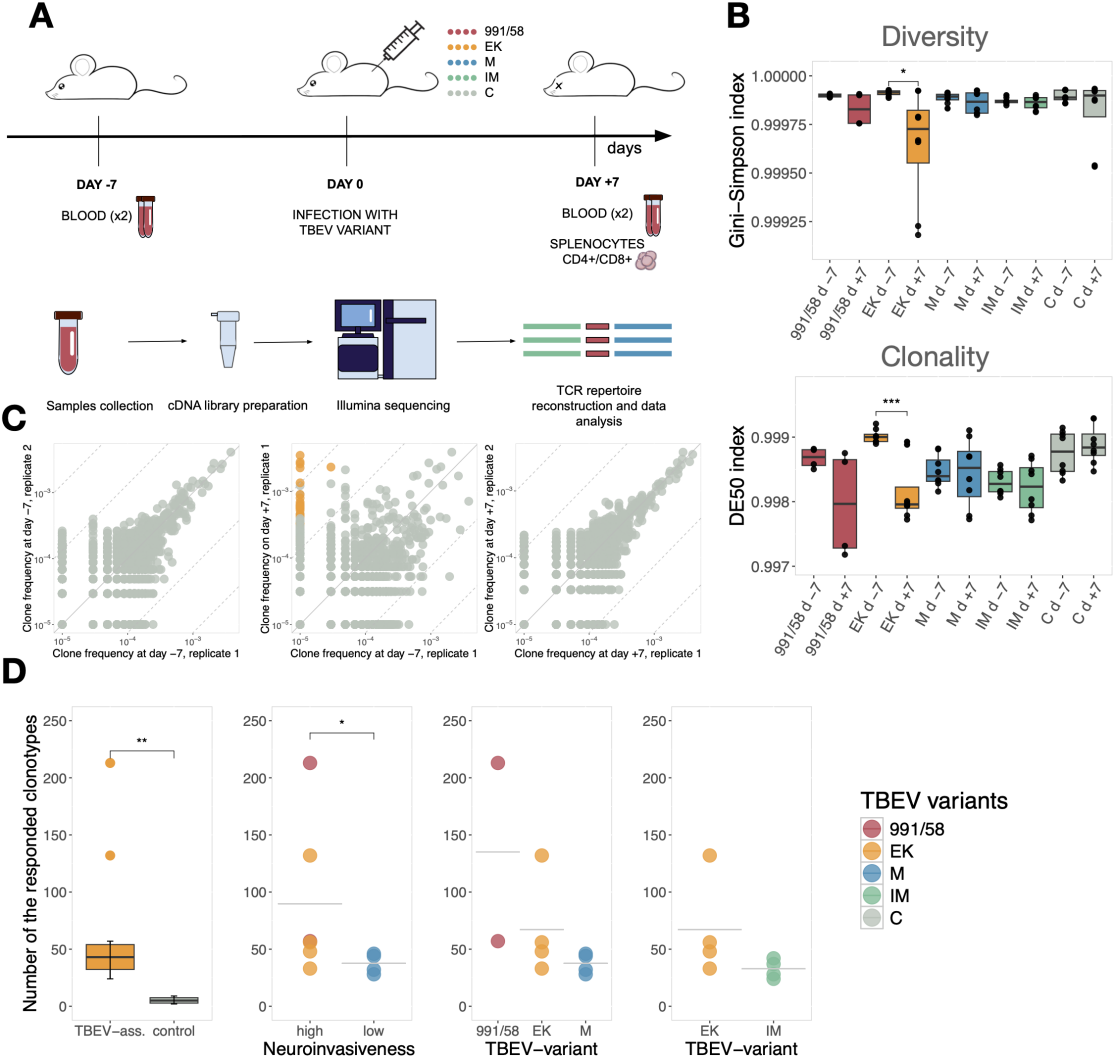
**(A)** Experimental design. Murine blood was taken before and after TBEV infection. Each mouse was infected with one viral variant (991/58, EK, M, IM) and analyzed separately. At both timepoints (day +7 and day -7) two biological replicates of whole blood were collected. Additionally, at day +7 splenocytes were separated into two fractions: CD4^+^ and CD4^-^. cDNA libraries were generated as described in Mouse TCR profiling kit (MiLaboratory LLC) and sequenced on Illumina platform. **(B)** TCRβ repertoire architecture. Diversity estimation Gini-Simpson index (top panel) and Clonality estimation DE50 index (bottom panel). Both metrics are presented for each mouse and are categorized based on the specific TBEV variant used for infection. Additionally, the data is organized by experimental day to facilitate comparison across different time points. Mann–Whitney test was carried out. Not significant not shown, *p < 0.05, ** p < 0.01, ***p < 0.001 **(C)** Relative abundance of TCRβ clones from a single mouse infected with the IM virus variant in repertoires from blood samples. Left and right panels show the relative abundance of a specific TCRβ sequence detected in replicas at a single day. The top panel refers to the repertoire at day -7, and the bottom panel to day +7. The middle panel represents the relative abundance of each TCRβ sequence in the same mouse at day -7 (x-axis) versus day +7 (y-axis). Clonotypes with significantly increased frequency are colored yellow. Axis shows clones concentration. Each dot represents a clonotype. Pseudocount is used for visibility. Dashed lines show concentration differences of 10 and 100-fold. **(D)** The number of significantly expanded clones for each mouse. First panel. Significant differences were observed in the number of responding TCRβ clonotypes between the control group and mice infected with TBEV. Second panel. Studied TBEV variants grouped by neuroinvasiveness. Third panel. Studied TBEV variants grouped by specific TBEV variant used for infection. Fourth. Two forms of the same virus: mature (EK) and immature (IM). Mann–Whitney tests were carried out. Not significant not shown, *p < 0.05, **p < 0.01, ***p < 0.001. Significant differences were observed in the number of responding TCRβ clonotypes between the control group and mice infected with TBEV (Mann–Whitney test, p = 0.003). Animals infected with high-neuroinvasive variants (991/58 and EK) had a higher number of responding TCRβ clonotypes compared to those infected with low-neuroinvasive (M variant)(Mann–Whitney test, p = 0.038).

We aimed to compare T cell immune response elicited by three virus variants (991/58, EK, and M) with differing levels of pathogenicity. The 991/58 and EK variants are highly virulent and neuroinvasive, in contrast to the low-virulent, low-neuroinvasive M variant. Additionally we compared the immune response to the mature (EK) and immature (IM) form of the same virus. We studied immune response by identifying specific T cell receptor sequences responding to each virus variant. Each group of mice (n=4) received a subcutaneous injection of one virus variant (**Figure 2A**). A control group consisting of four animals was injected with PBS. The characteristics of the virus dose used to infect animals are given in the table (**Table 2**). Blood samples were collected from all mice seven days prior to infection (day -7), and each sample was divided into two replicates for parallel processing. At day +7 after virus inoculation (day 0), all mice were bled again (two replicates) and sacrificed to harvest the spleen. Splenocytes were separated into CD4^+^ and CD8^+^ T cell subpopulations. Total RNA from whole blood and splenocyte CD4^+^ and CD8^+^ fractions was isolated and used for TCRβ library preparation.

Although the blood draw samples for a mouse are very small (typically around 100 μl) we were able to identify on average 48,366 (median = 43526, range 19,726–105,848, n = 40) TCRβ clonotypes per sample replicate taken at day -7 and 43,250 (median = 42400, range 78–101,342, n = 40) TCRβ clonotypes at day +7. CD4+ T cells from the spleen contain on average 79,796 (median = 71508, range 44,482–217,039, n = 20) clonotypes while CD8+ fraction 77,416 (median = 73970, range 38,717–139,784, n = 20) TCRβ clonotypes. Unfortunately, reconstructed day +7 repertoire of two mice infected with 991/58 variant contained insufficient number of clones to detect any alterations (**Supplementary Figure 2**). These mice were excluded from further analysis. To enable comparative analysis of TCR repertoire architecture, sequencing reads were normalized across samples. Repertoire diversity was quantified using the Gini-Simpson index, and clonality was assessed via the DE50 metric (**Figure 2B**). The results show that the values for both metrics are close to 1 indicating highly diverse repertoires with balanced clonotype frequencies, reflecting high population evenness. With the exception of the EK variant, no significant changes in diversity or clonality were observed. The infection with EK variant exhibited a significant reduction in both diversity and evenness, indicative of an antigen-driven immune response characterized by the clonal expansion of specific T cells. To investigate potential minor changes in repertoire architectures that were not captured by these broad metrics, further in-depth analysis was performed.

The identification of virus-responding clonotypes for each mouse was performed using a previously developed approach (12). TCRβ repertoires from blood replicates of the same day showed strong convergence, demonstrating high reproducibility. Data for single mouse infected with IM is shown (**Figure 2C**, left and right panel). This reproducible data allowed for a comparison of TCRβ repertoires from whole blood between day -7 and day +7 using EdgeR software. The normalized frequency of each clonotype was calculated at 7 days before and at 7 days after infection, a statistical Generalized Linear Model was employed to assess the probability of clone expansion in response to viral infection. We determined the TCRβ clonotype was expanded only if its concentration significantly changed after infection (Materials and Methods). Every mouse underwent individual analysis according to this approach (**Supplementary Figure 5**). Some expanded clones were undetectable at day -7, while the abundance of the others drastically increased after TBEV-infection (**Figure 2C**, middle panel).

**Figure 3:**
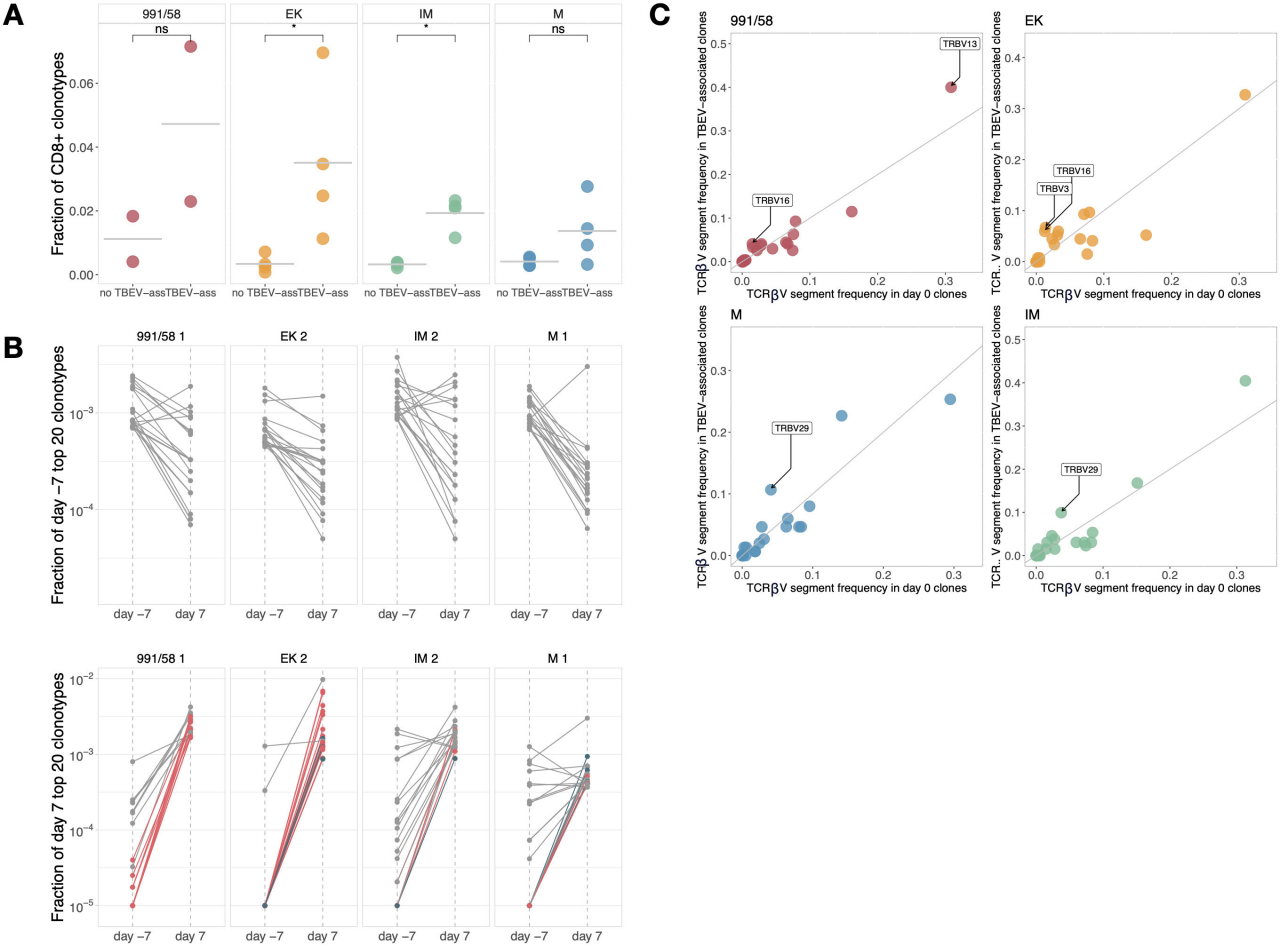
**(A)** The proportion of CD8^+^ expanded TCRβ clonotypes for each animal. This proportion was calculated by summing the fractions of CD8^+^ responding clones for each mouse independently. The T cell response is predominantly shifted towards CD8^+^ cells across all TBEV variants. Notably, a statistically stronger shift towards CD8^+^ dominance was observed in the EK and IM groups, as confirmed by Wilcoxon tests (*p = 0.03 for both). **(B)** Dynamic of the top repertoire. Fraction of twenty the most abundant clones at day -7 and their fraction at day +7 (top panel). Fraction of twenty the most abundant clones at day +7 and their fraction at day -7 (bottom panel). Grey color represents clones without TBEV-association. TBEV-associated CD8^+^ clones colored in pink, CD4^+^ in blue. **(C)** Comparison of V-segment frequency between TBEV-associated clones and the intact repertoire. Labels indicate V segments that are statistically more prevalent among TBEV-associated clones for each viral variant, highlighting specific V segments favored in the immune response to different variants of TBEV.

To analyze the magnitude of the T cell response, we compared the number of the detected TCRβ clonotypes between groups of mice infected with different TBEV variants. The number of such clonotypes varied from 24 (IM variant) to 213 (991/58 variant) for infected animals, and from 2 to 9 for the control animals. Infected animals exhibited a greater number of expanded clonotypes compared to the control group (Mann–Whitney test, p = 0.003) (**Figure 2D**, first panel). For the selected comparison group we combined data from mice by virulence and neuroinvasiveness. A statistically significant difference was observed in the number of expanded TCRβ clonotypes between animals infected with the low-virulent M-variant and those infected with the high-virulent TBEV variants (991/58 and EK) as determined by Mann–Whitney tests (p = 0.038) (**Figure 3D**, second panel). Analysis of each variant independently shows the same trend (**Figure 2D**, third panel). No statistically significant differences in the magnitude of the T cell response were recorded between the mature and immature forms of the viruses. However, a trend toward an increased number of responded T cell clonotypes to the mature form of the EK virus was detected (**Figure 2D**, fourth panel).

Thus, we show that TBEV infection induces proliferation of a specific group of TBEV-associated T cells, and infection caused by high-virulent and high-neuroinvasive TBEV variants induce stronger T cell response than infection caused by low-virulent and low-neuroinvasive TBEV variants.

#### 3.2.2 Characterization of virus responding clonotypes

In order to identify CD4^+^/CD8^+^ phenotype of the TBEV-associated clonotypes we used magnetic separation of splenocytes at day +7. Based on the presence of the responding clonotype sequence in CD4^+^ or CD4^-^ TCRβ repertoire generated from spleen T cells, we assigned each expanded clone to CD4^+^ or CD8^+^ T cell subset (**Supplementary Figure 6**). In examining the shift towards a cytotoxic T cell response, we calculated the average count of CD8^+^ cells by comparing the non-TBEV-specific TCRβ repertoire of equivalent size to that of the TBEV-associated repertoire for each mouse individually (**Figure 3A**). This approach allowed us to assess whether there was an enrichment of CD8^+^ cells in response to TBEV infection relative to background levels, thereby providing insights into the role of cytotoxic T cells in this context. Our results reveal an increased presence of CD8^+^ cells within the TBEV-associated repertoires. Statistical significance was observed exclusively for TCRβ clonotypes associated with the EK and IM variants, as determined by Wilcoxon tests (p = 0.03 for both). This finding suggests a trend towards preferential recruitment and expansion of cytotoxic CD8^+^ T cells towards virus-infected cells.

We observed significant alterations in the repertoire structure within the most abundant TCRβ clonotypes following TBEV infection (**Figure 3B**). Within the top 100 most abundant clones at day -7 none were identified as TBEV-associated. By the day +7 these clones exhibited little changes in their abundance levels and largely remained among the top of the TCRβ repertoire. These clonotypes represent the overall stability of TCR repertoire. In contrast, by day +7 post-infection, the top repertoire of infected mice contained between 21 and 72 novel expanded clones, whereas only between 2 and 9 expanded clones were observed in the control group (Mann–Whitney, p = 0.03). Notably, some of the most frequent clones at day +7 had low concentrations or were undetectable at day -7. In conclusion, these findings suggest that strong immunogenic stimuli like TBEV infection can profoundly impact the range of T cell clones within a given repertoire.

The selection of particular V genes in TCR sequence has been observed in response to immune challenges (8, 14, 56–59). To further evaluate the structural characteristics of virus-responding clones, we performed an analysis of V-segment frequency among these clonotypes within groups of animals infected with different TBEV variants. For each group of mice, we computed the frequency of each V-segment among the TBEV-associated clones and among total T cell clone repertoire (**Figure 3C**, **Supplementary Spreadsheet 2**). Our findings indicate a statistically significant increase in the utilization of TRBV13 and TRBV16 for mice infected with the 991/58 virus variant, TRBV3 and TRBV16 for EK, and TRBV29 for mice infected with M or IM variants. We consistently observed a pattern in responding TCRβ clonotypes to neuroinvasive 991/58 and EK TBEV variants utilizing TRBV16. Surprisingly we did not find any similarities in V-segment preferences for mature EK and its immature form IM. These observations may indirectly imply a similarity in TCR sequences that respond to distinct TBEV variants associated. It also suggests that different epitopes might be involved in triggering T cell reactions for these variants, potentially influencing the nature of the immune response and disease outcomes.

#### 3.2.3 Virus responding clones show convergence in CDR3 sequence

**Figure 4:**
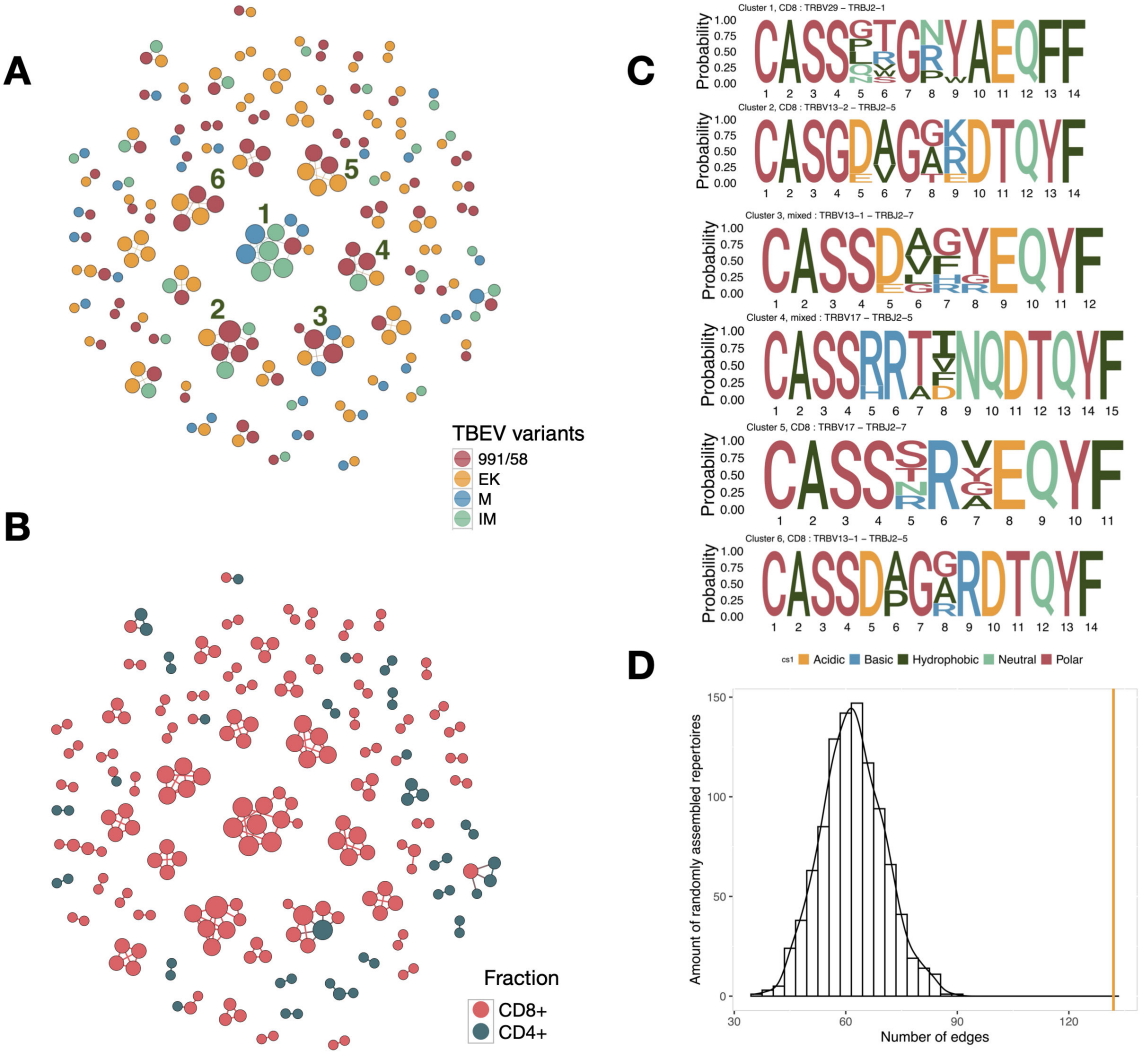
**(A)** Similarity network of TCRβ CDR3 amino acid sequences associated with TBEV. Clusters with less than 2 members are not shown. Colors represent specificity to TBEV variants: 991/58 (red), EK (yellow), M (blue), IM (green) **(B)** Similarity network of TCRβ CDR3 amino acid sequences associated with TBEV. Colors represent CD8^+^ (pink) and CD4^+^ (blue) fractions. **(C)** CDR3 amino acid sequences of the TBEV-associated clonotypes from the indicated clusters are presented as sequence logos. Cluster numbers, phenotypes (CD4^+^ or CD8^+^), and V-J segments of T cell clones are shown on the top of the logos. Amino acids are colored according to their chemical properties. **(D)** Edge counts from generated random TCRβ samples at day -7. The yellow line represents the edge count observed in the similarity network of TCRβ CDR3 amino acids associated with TBEV infection.

In order to identify structural similarities among TBEV responding T cells we constructed TCRβ CDR3 amino acid sequence similarity networks for all clonotypes expanded after TBEV infection (**Figure 4A**). The largest cluster (#1) includes 10 CDR3 sequences identified in response to each of the investigated TBEV variants. These sequences are characterized by TRBV29 and TRBJ2-1. We also observed two clusters of 7 TCRβ CDR3 sequences formed by clones characterized for 3 TBEV variants. Our analysis revealed that clusters containing 4–5 CDR3 sequences were composed of TCRβ clonotypes that are specific to the 991/58 and EK variants.

The majority of TBEV-specific T cells, whose TCRβ CDR3 sequences form clusters, are CD8^+^ cells. Notably, the largest cluster identified consists entirely of CD8^+^ T cells (**Figure 4B**). This clustering pattern suggests that these CD8^+^ T cell populations play a significant role in antiviral activity against TBEV. We also identified amino acid consensus motifs within the TCRβ CDR3 of the found clustered TBEV-specific T cells (**Figure 4C**). We computed the probability of generation (P_gen_) for TBEV-associated TCRβ amino acid sequences to evaluate their likelihood during V(D)J recombination. We observed a substantial range in P_gen_ values across the TBEV-associated TCRβ sequences, moreover TCRβ sequences, which are forming clusters, are characterized by P_gen_ 10^–5^ to 1.3×10^-9^. This broad distribution indicates that TBEV-associated TCRβ amino acid sequences from clusters are statistically common products of V(D)J recombination, indicating them as “public” clones (**Supplementary Figure 7**). Clusters with higher P_gen_ values contribute to the public TCR repertoire, but the likelihood of random (independent) generation of such receptors across different thymocytes or organisms remains relatively low (53). The appearance of considerable quantities of clones carrying these TCRβ sequences requires selective processes and clonal expansion, which are driven by antigen recognition and immune response dynamics.

To demonstrate that the identified TCRβ CDR3 clusters were formed as a result of TBEV infection rather than being a feature of the TCR repertoire, we randomly extracted TCRβ clonotypes from day -7 samples of each mouse. The number of clones extracted was equivalent to the size of the TBEV-associated TCRβ repertoire. We then calculated the number of edges formed by their amino acid sequences. This process was repeated 1,000 times (**Figure 4D**). The analysis revealed that the TBEV-associated TCRs formed clusters containing more than twice the average number of clusters observed in the randomized day -7 repertoires, supporting their association with the infection.

We also conducted an analysis of published data on murine TCR amino acid sequences expanded following infection with another TBEV strain (54). Fujii et al. infected C57BL/6 mice with Oshima 5–10 strain. On day 13 post-infection RNA was isolated from the brain and spleen. Nucleotide sequences of TCR were obtained with the use of specific primers annealed to three only V-segments and constant region, afterwards cloned and sequenced by Senger method. Authors observed an increased level of certain V-genes in the brain of infected mice, also identified differences in V-gene families’ frequencies from spleen samples between dying and surviving mice. Amino acid sequences of TCR CDR3 regions of cDNA clones derived from TBEV-infected mouse brains are published by the authors. In order to find similarities between our and published data we looked for exact matches, afterwards matches with Hamming distance 1 and 2. However, no similarities were identified between these sequences and those observed in our study (**Supplementary Figure 8**).

## 4 Discussion

The progression of TBEV infection is influenced by the interplay between viral and host determinants, particularly the adaptive immune response. This response is characterized by virus-specific T cell activity, which plays a crucial role in controlling viral pathogenesis. Aregay and co-authors have demonstrated that the magnitude of the early virus-specific T cell response inversely correlates with TBE disease severity, highlighting the importance of robust T cell immunity in controlling viral pathogenesis (60).

In this study, we employed high-throughput sequencing of TCRβ repertoire to identify and characterize alterations in T cell population during the response to TBE viral variants derived from a single parental strain. Using a murine model of infection with TBEV variants, we investigated three viral variants with distinct pathogenic profiles: EK-328 and 991/58, which are characterized by high neuroinvasiveness, high virulence and elevated mortality rates and M-variant, which display low neuroinvasiveness, low virulence and reduced mortality rates. All virus variants used in this study differ by no more than a few amino acid substitutions in structural and non-structural proteins. In addition, we used an immature (IM) virus that did not differ from the EK-328 strain in the amino acid sequence of viral structural and non-structural proteins, but contained the prM protein in the virion. The immature virus is non-infectious; however, when obtained by the addition of acidotropic amines during culture, a small proportion of mature and partially mature infectious virus particles are present in the population; these mature particles play the main role in virulence and mortality rate for IM virus.

Substitutions in non-structural proteins (e.g., NS2a and NS4a in 991/58) may introduce novel epitopes, further shaping T cell clonal responses. Conversely, the M variant, characterized by lower virulence (**Table 1**), may exhibit limited antigen presentation. This restriction is attributed to structural differences, specifically, mutations in the E protein of the M variant, which does not differ from variant 991/58 in nonstructural proteins. The presence of the prM protein in non-infectious virions of the IM virus or a reduced capacity for viral replication may influence differences in the T-cell immune response compared to EK. The main complexity in identifying TBEV-associated clones is the lack of known immunogenic epitopes (61). To overcome this challenge, we analyzed data from two biological replicates at two time points (before and after infection). Despite contemporary trends, pooling samples is not a viable approach for tracking individual T cell responses (62). Therefore, each sample from each mouse was processed independently to ensure accurate analysis. Our findings indicate that infection with any of the studied TBEV variants results in significant alterations in the TCRβ repertoire through massive T cell expansion, with some T cell populations expanding over 4000 fold after infection. We identified several dozens of unique TCRβ clonotypes in each infected mouse that expanded in response to TBEV infection, whereas the control group did not exhibit significant repertoire changes. In total, we identified 820 TBEV-associated T cell clones from 14 animals infected with four distinct virus variants.

This expansion reflects antigen-driven selection – virulent variants like EK-328 and 991/58 induce high virulence, promoting robust epitope presentation and recruiting higher T cell number. On the other hand, M variant’s E protein substitutions reduce conformation stability (28). While immature particles may delay adaptive responses, shifting T cell dynamics to later stages. Nevertheless, even variant with milder disease manifestations (M) triggered sufficient clonal activation to control infection, albeit with fewer responding clones. Previously studied T cell dynamics during SARS-CoV-2 diverged between severe and mild forms: severe disease exhibits lymphopenia and exhaustion (63), while mild form is characterized by increased T cell numbers and their transition to memory states (64). We observed little difference in the magnitude of TCRβ clonotypes expansion among the TBEV variants. However, there was a notable increase in the number of responding clones corresponding to the high-virulence and high-neuroinvasiveness of the TBEV variants (991/58 and EK) compared to variant with low-virulence and low-neuroinvasiveness (M variant).

For the most part T cell response against every TBEV variant consists of CD8^+^ cells. The role of CD8^+^ T cells in the context of TBEV infection is particularly noteworthy. These cells are responsible for the elimination of infected cells, on the other hand, studies utilizing severe combined immunodeficiency mouse models have demonstrated the involvement of CD8^+^ T cells in immunopathogenesis (65). Notably, we observe a statistically stronger shift towards CD8^+^ phenotype for EK-328 and IM viruses, but not for TCRβ associated with M variant. Although, the common trend towards increased fraction of CD8+ TBEV-associated clones can be seen. Small sample sizes may affect p-values. We associate the lower M-variant-associated CD8^+^ T cells abundance with reduced virulence and diminished ability to infect host cells, due to mutations in E protein. The loss of highly immunogenic epitopes from the E protein may occur, leading to a reduced expansion of specific CD8^+^ T cells. This outcome may be attributed to the fact that M variant induces infection without the manifestation of clinical symptoms.

The individual structure of the TCR repertoire is organized into distinct clusters based on sequence similarity (20, 66). Although the T cell repertoire is highly individual, even among mice of the same inbred strain, we were able to demonstrate a notable similarity in TBEV-responding clones across mice infected with different viral variants. Previous attempts have been made to assess the similarities TCR structure of brain infiltrating T cells during TBEV infection with the Oshima strain (54). Through graph analysis, we observed predominance of TCRβ clusters among clones associated with the EK variant, as well as between the EK-328 and 991/58 groups. Despite this fact the largest cluster includes clones responding to different TBEV variants. The TBEV-associated TCRβ CDR3 sequences within this cluster are characterized by the use of TRBV29, a Vβ gene segment frequently observed among TCRs specific to the IM and M variants. This suggests the presence of a conserved viral epitope shared between all four observed TBEV variants, which lacks mutations and is recognized by these clustered TCRβ CDR3. EK-328 and 991/58 share non-structural protein substitutions, possibly fostering cross-reactive responses. However, M variant’s E protein mutations likely alter epitopes, reducing TCR sequence cluster overlap and underscoring how minor amino acid changes disproportionately impact TCR recognition.

In this study, we identified TBEV-specific T cell clones by analyzing changes in the TCR repertoire driven by clonal expansion in response to the virus. However, this approach has significant limitations. It is antigen-agnostic and does not reveal the specific epitopes recognized by these TCRs. Currently, no immunodominant epitopes for TBEV have been identified, which hinders our ability to validate these findings using MHC multimer staining or peptide stimulation assays. Additionally, detecting robust immune responses in mice presents challenges due to the limited sample size, meaning the identified TCRs likely represent only a subset of the overall immune response. Our analysis was further restricted to peripheral blood and spleen tissues, as we did not assess the TCR repertoire across other tissues. Future studies addressing this limitation would provide a more comprehensive understanding of the immune response to TBEV.

## 5 Conclusions

Overall, our findings provide valuable insights into the mechanisms underlying the immune response to TBEV enabled us to detect even minor alterations and quantify their impact on the immune response. Obviously, further more detailed study of the role of TCR in the process of neuroinfection caused by TBEV is required.

## Data Availability

The data presented in the study are deposited in the SRA repository, accession number PRJEB91685. The data can be found at https://www.ncbi.nlm.nih.gov/bioproject/PRJEB91685.
