# Supplementary material for: Tick-borne encephalitis virus variants drive distinct TCR repertoire alterations
Source: Front Immunol. 2025 Dec 18;16:1663781. doi: 10.3389/fimmu.2025.1663781 (PMC12756428; doi:10.3389/fimmu.2025.1663781)
Supplement: Supplementary file 3 [file SupplementaryFile1.docx]

Supplementary Material

# Supplementary method

## TCRβ cDNA Library Preparation and Sequencing

Total RNA was extracted from all samples using TRIzol reagent, following the manufacturer's guidelines, and was subsequently eluted in 10 μl of RNase-free water. An equal amount of isolated RNA from each sample was utilized in the cDNA synthesis reaction. Each cDNA synthesis reaction was purified with Ampure XP beads (Beckman Coulter, USA). The resulting first-strand cDNA was subjected to the first PCR, which consisted of 21 cycles. The PCR products were then purified using Ampure XP beads (Beckman Coulter, USA) and eluted in 30 μl of elution buffer. For the second round of PCR, 1 μl of the first PCR product served as a template and underwent 15 to 18 cycles. Finally, the samples were combined and purified again using Ampure XP beads (Beckman Coulter, USA).

# Supplementary Figures and Tables





Supplementary Figure 1. Passage history of M variant and its revertant 991/58

**Supplementary Table 1.** Nucleotide substitutions in the genomes of revertant 991/58 and M variant in comparison with the parental strain EK-328

| region of the genome | genome position | EK-328 | variant M | variant 991/58 |
| --- | --- | --- | --- | --- |
| 5’-UTR | 19 | A | G | A |
| 5’-UTR | 42 | C | A | A |
| Signal sequence prM | 470 | C | T | T |
| protein E | 843 | T | T | С |
| 122 AA^*^ of protein E | 1337 | A | G | A |
| 124 AA of protein E | 1342 | A | A | G |
| protein E | 1605 | G | G | A |
| protein E | 2190 | C | T | T |
| 426 AA of protein E | 2249 | C | Y | C |
| protein E | 2362 | T | C | C |
| NS1 | 3300 | G | C | G |
| NS1 | 3387 | C | T | T |
| NS1 | 3438 | G | A | A |
| 52 AA of protein NS2A | 3672 | G | T | T |
| NS3 | 4827 | G | A | A |
| NS3 | 5515 | C | C | T |
| 22 AA of protein NS4A | 6526 | A | G | G |
| 41 AA of protein NS4A | 6584 | G | A | A |
| NS4B | 7278 | C | C | T |
| NS4B | 7437 | G | C | C |
| NS5 | 8574 | G | R | G |
| NS5 | 9888 | T | C | C |
| 3’-UTR | 10104 | C | T | C |

* Amino acid


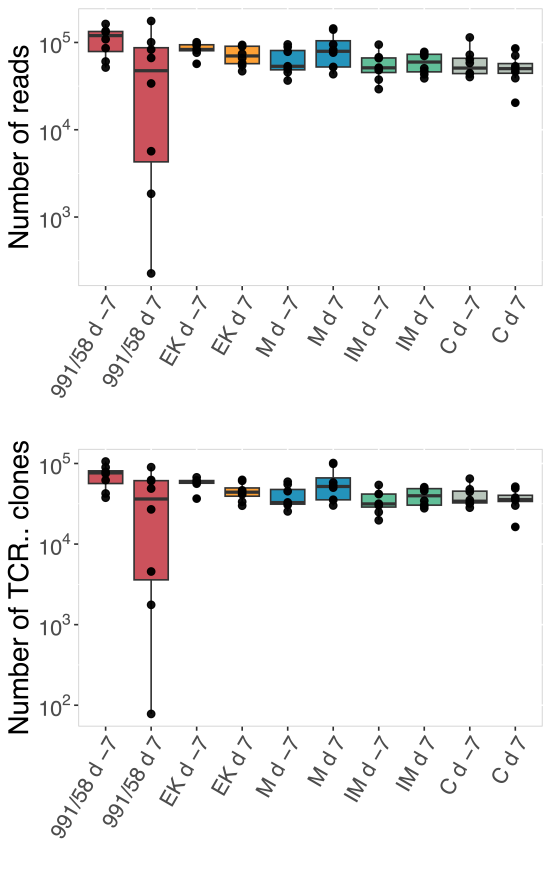


**Supplementary Figure 2**. The number of reads (top panel) and the number of unique clones (bottom panel). Both metrics are presented for each mouse and are categorized based on the specific TBEV variant used for infection. Additionally, the data is organized by experimental day to facilitate comparison across different time points.


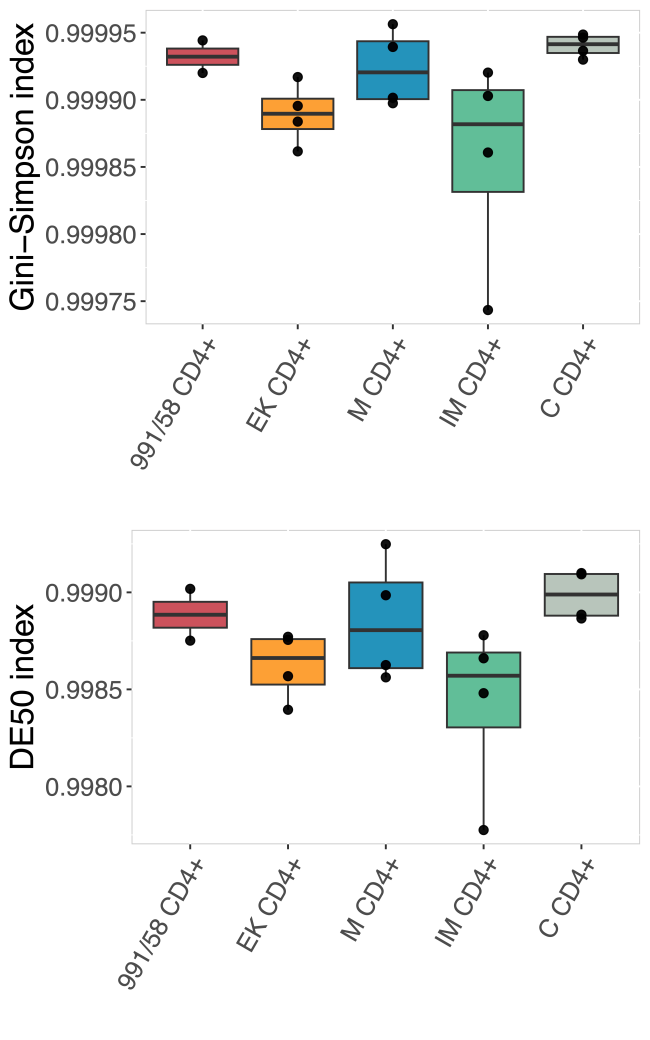

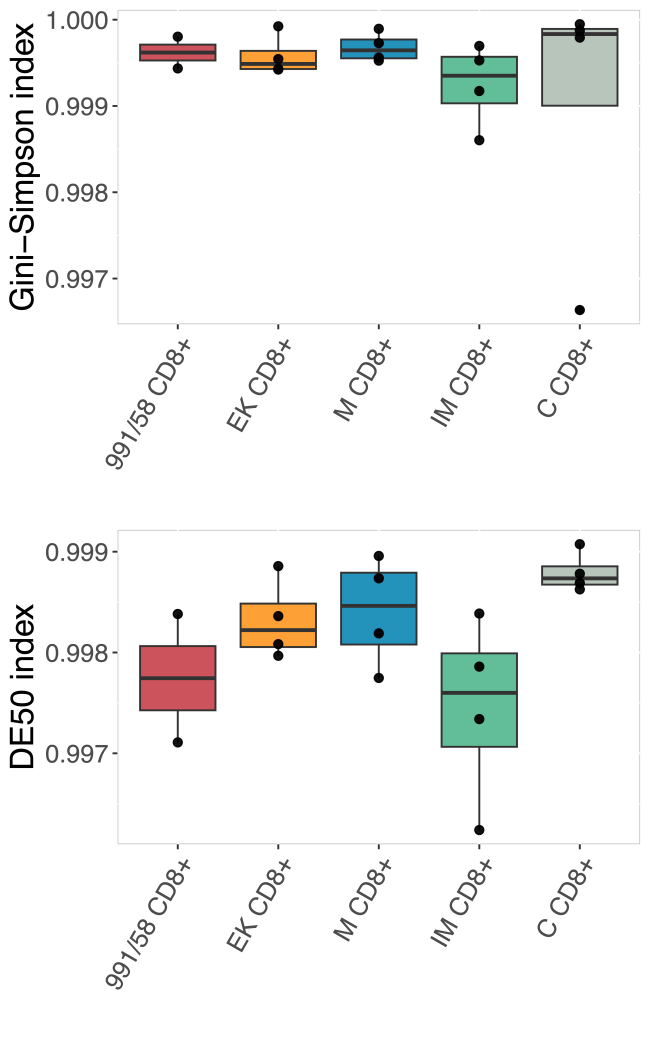


**Supplementary Figure 3**. Diversity Gini-Simpson index (top panels) and Clonality DE50 index(bottom panels) for spleen samples. Both metrics are presented for each mouse and are categorized based on the specific TBEV variant used for infection. Additionally, the data is organized by CD8/CD4.


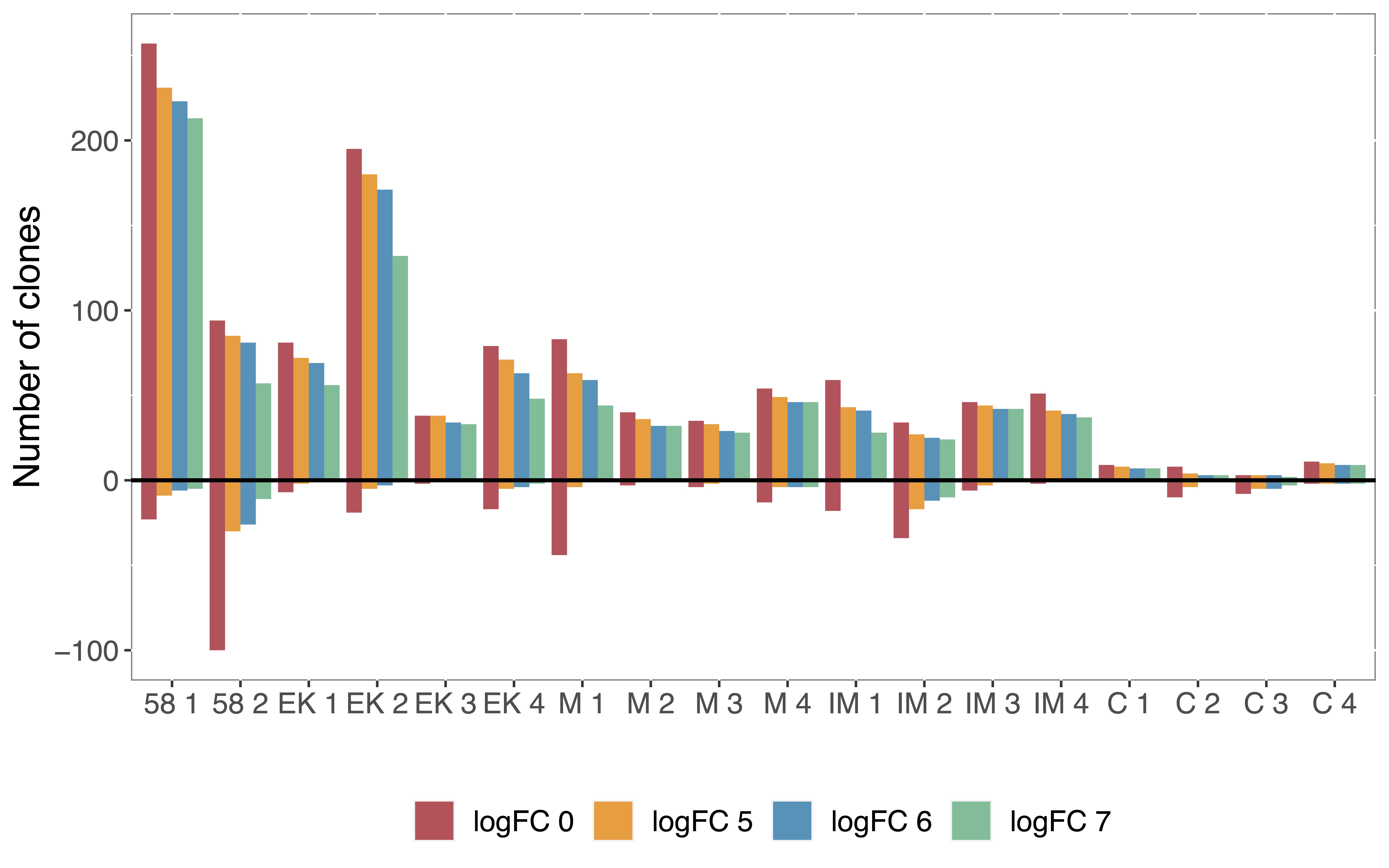


**Supplementary Figure 4**. Threshold selection for determining TCR clonotypes expanded


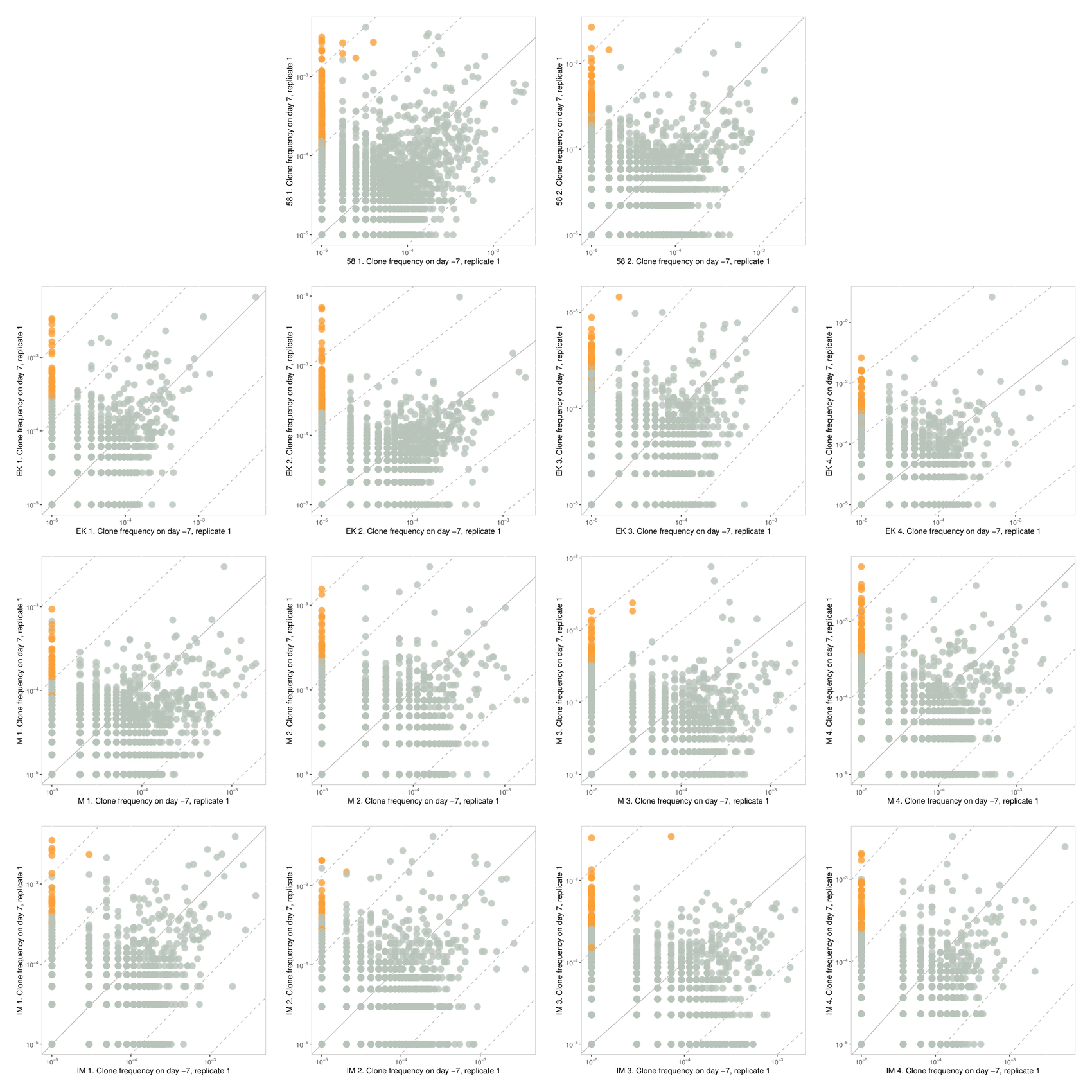


**Supplementary Figure 5**. Relative abundance of each TCRβ sequence in the same mouse at day -7 (x-axis) versus day +7 (y-axis). Clonotypes with significantly increased frequency are colored yellow. Axis shows clones concentration at different days for one replicate. Each dot represents a clonotype. Pseudocount is used for visibility. Dashed lines show concentration differences of 10 and 100-fold


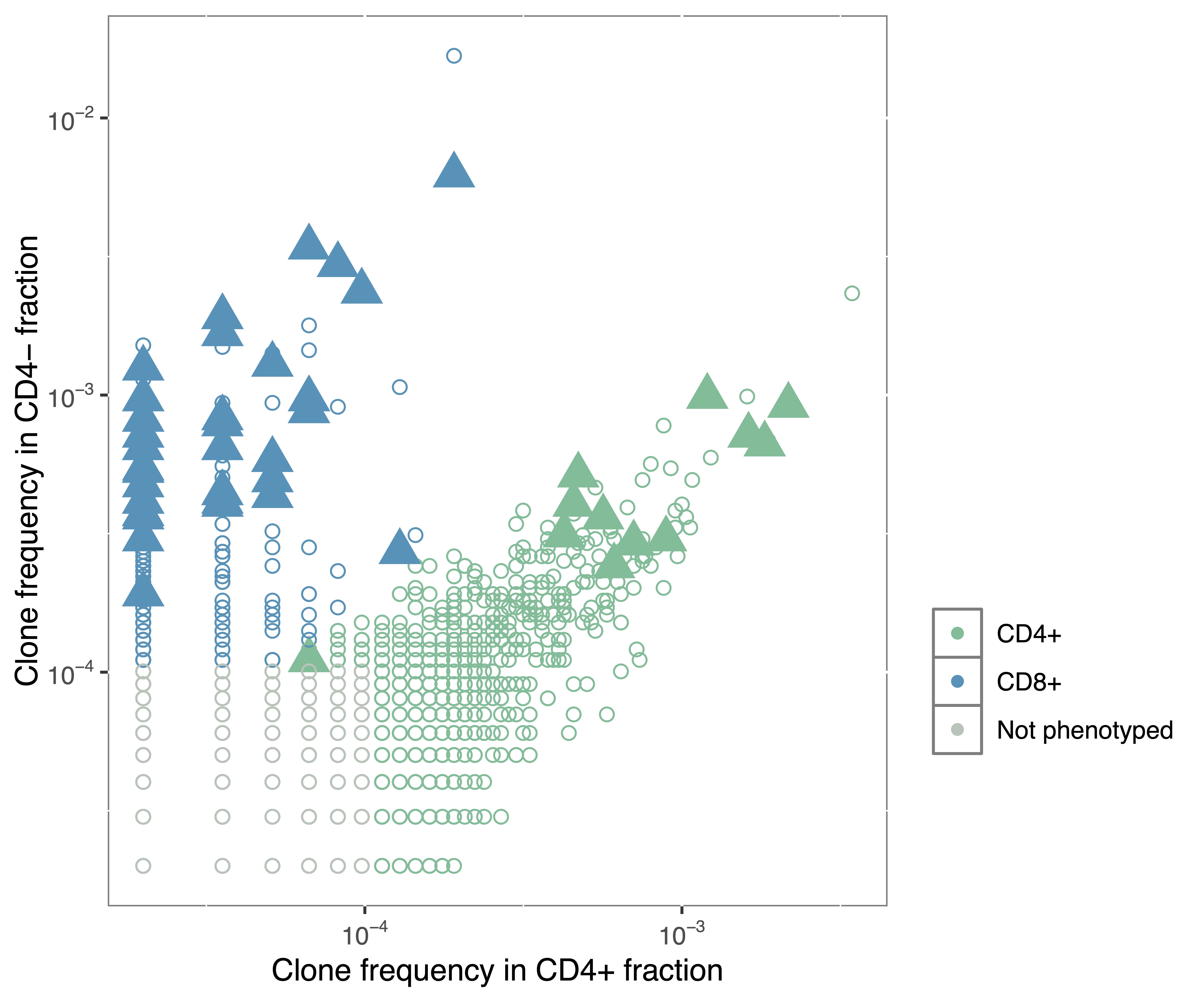


**Supplementary Figure 3**. Phenotyping of the expanded clones using repertoires from magnetic separated splenocytes. The relative abundance of the clonotype in CD4+ fraction (x-axis) is plotted against the relative abundance in the CD4- fraction (y-axis) extracted from splenocytes at day +7. Clonotype is determined as CD8+ if its concentration is higher than 10-4 and presented in CD4- fraction over 3 times more than in CD4+ fraction. All clonotypes phenotyped as CD8 are coloured in blue. Clonotype is determined as CD4+ if its concentration is higher than 10-4 and presented in CD4+ fraction over 3 times more than in CD4- fraction. All clonotypes phenotyped as CD8 are coloured in green. Triangles represent expanded clonotypes. For every mouse we managed to distinguish phenotype of all expanded clonotypes.

**Supplementary Table 2**. Number of CD4+/CD8+ clones per mouse in different fractions.

|  |  | Count of CD4+ clones | | | Count of CD8+ clones | | |
| --- | --- | --- | --- | --- | --- | --- | --- |
| TBEV variant | Mouse id | expanded | day -7^A^ | expanded at day -7^A^ | expanded | day -7^A^ | expanded at day -7^A^ |
| 991/58 | 991/58 1 | 72 | 109.37 | 0.00 | 141 | 103.45 | 1.25 |
| 991/58 | 991/58 2 | 10 | 33.79 | 0.00 | 47 | 23.70 | 0.15 |
| EK | EK 1 | 8 | 35.92 | 0.00 | 48 | 19.37 | 0.00 |
| EK | EK 2 | 33 | 91.18 | 0.00 | 99 | 40.82 | 0.04 |
| EK | EK 3 | 5 | 25.60 | 0.00 | 28 | 7.94 | 0.00 |
| EK | EK 4 | 12 | 34.89 | 0.00 | 36 | 13.31 | 0.00 |
| IM | IM 1 | 5 | 18.17 | 0.00 | 23 | 9.43 | 0.08 |
| IM | IM 2 | 9 | 13.18 | 0.00 | 15 | 11.16 | 0.05 |
| IM | IM 3 | 11 | 23.93 | 0.00 | 31 | 18.08 | 0.09 |
| IM | IM 4 | 3 | 23.56 | 0.00 | 34 | 12.73 | 0.00 |
| M | M 1 | 28 | 25.05 | 0.00 | 16 | 18.90 | 0.00 |
| M | M 2 | 15 | 18.37 | 0.00 | 17 | 13.19 | 0.00 |
| M | M 3 | 9 | 16.92 | 0.07 | 19 | 11.29 | 0.03 |
| M | M 4 | 21 | 22.55 | 0.00 | 25 | 23.44 | 0.00 |

1. The number of CD4+/CD8+ clones were calculated as an average number of CD4+/CD8+ counted from 100 times repeated random clones repertoire size of expanded repertoire for each mouse individually.


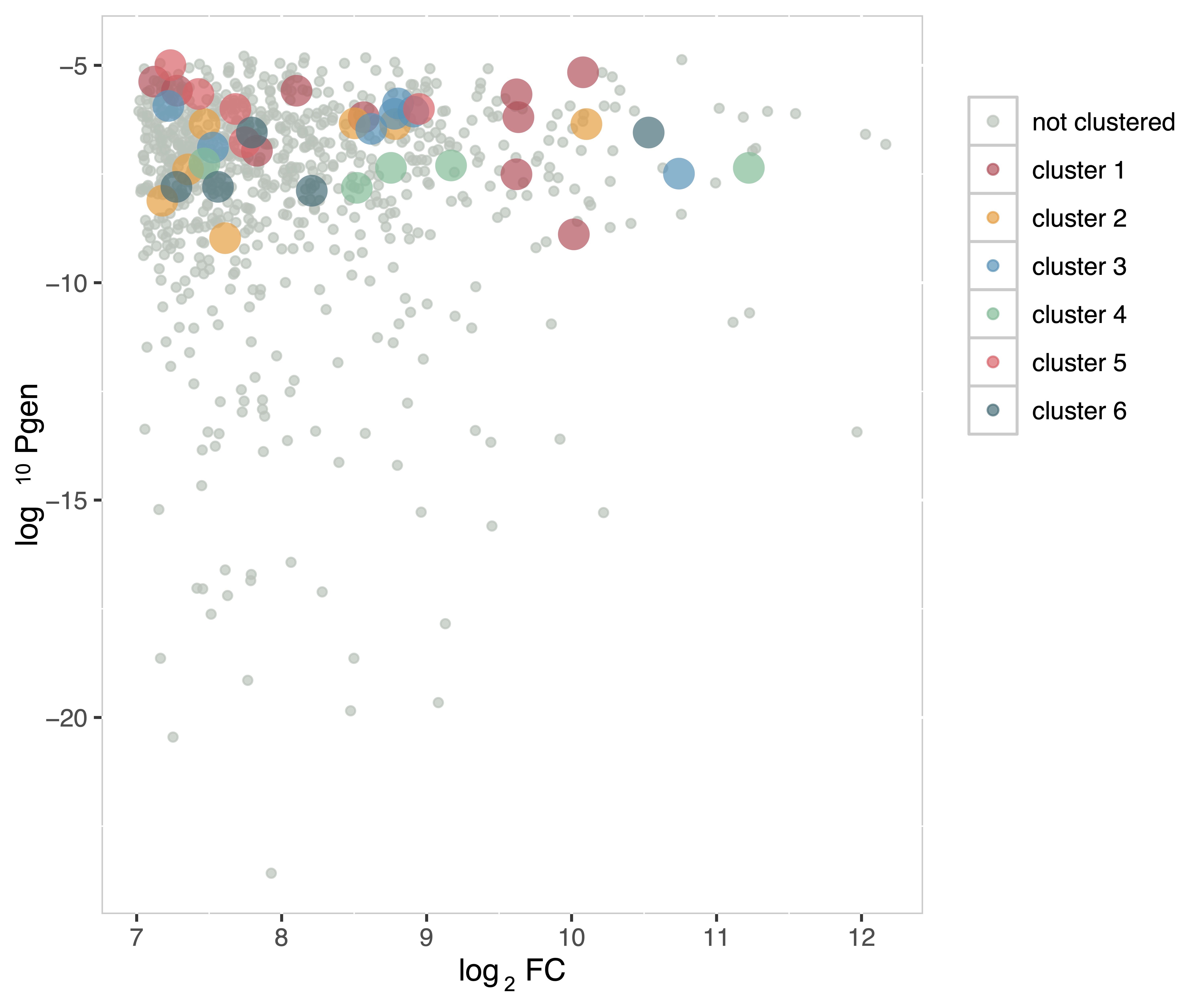


**Supplementary Figure 4**. The increase rate and probability of generation for TBEV associated clonotypes. Big dots represent clustered clonotypes, color represents the cluster number.


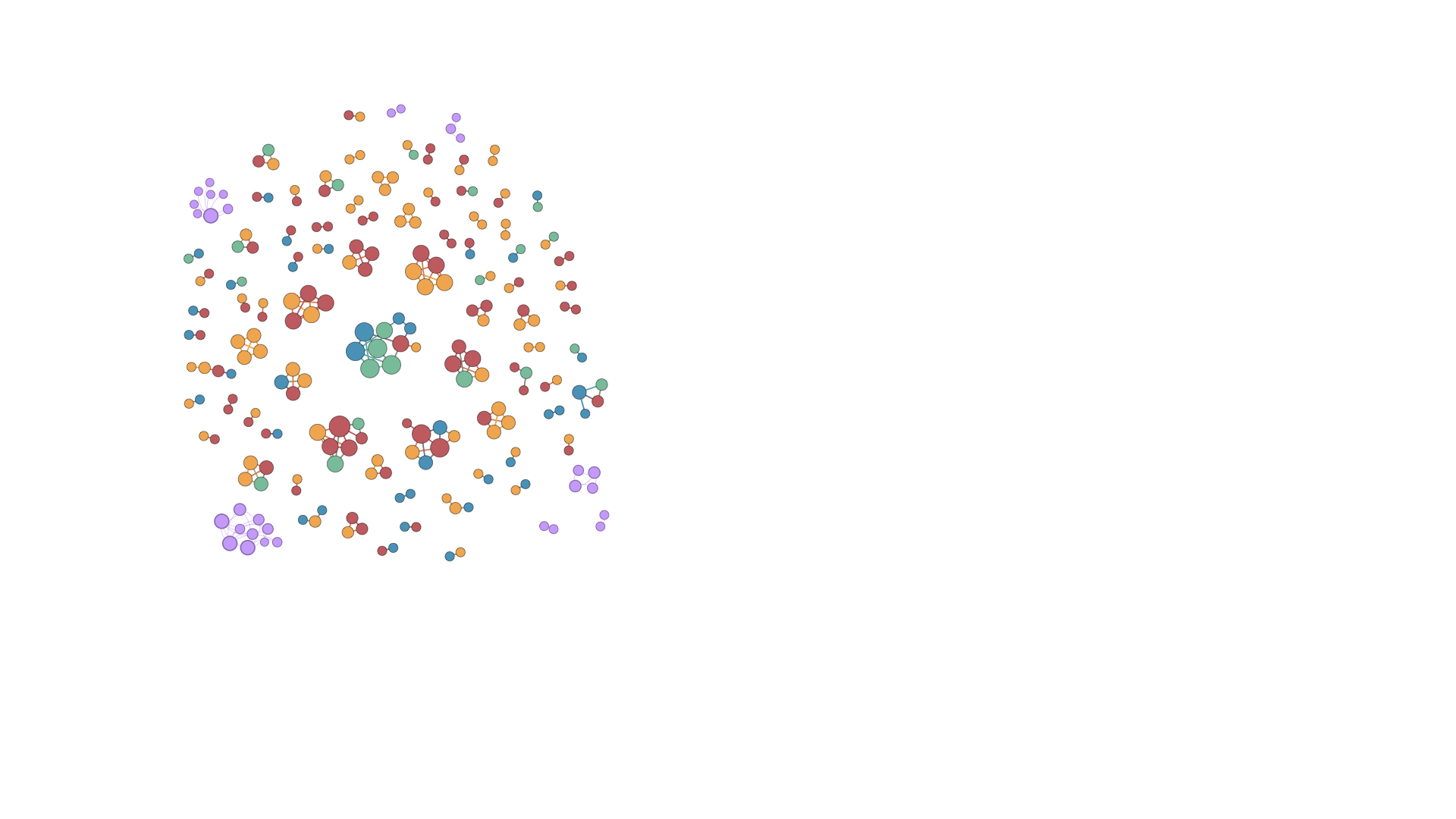


**Supplementary Figure 5.** Similarity network of TCRβ CDR3 amino acid sequences associated with TBEV. Each dot represents a clonotype, edges connect sequences with Hamming distance 2 or less. Clusters with less than 2 members are not shown. Colors represent specificity to TBEV variants: 991/58 (red), EK (yellow), M (blue), IM (green) and to Oshima strain (purple).
